# Extremophilic *Natrinema versiforme* Against *Pseudomonas aeruginosa* Quorum Sensing and Biofilm

**DOI:** 10.3389/fmicb.2020.00079

**Published:** 2020-02-06

**Authors:** Tunahan Irmak Başaran, Didem Berber, Barış Gökalsın, Annabella Tramice, Giuseppina Tommonaro, Gennaro Roberto Abbamondi, Merve Erginer Hasköylü, Ebru Toksoy Öner, Carmine Iodice, Nüzhet Cenk Sesal

**Affiliations:** ^1^Department of Biology, Institute of Pure and Applied Sciences, Marmara University, Istanbul, Turkey; ^2^Department of Biology, Faculty of Arts and Sciences, Marmara University, Istanbul, Turkey; ^3^Institute of Biomolecular Chemistry-CNR, Pozzuoli, Italy; ^4^Department of Bioengineering, Institute of Pure and Applied Sciences, Marmara University, Istanbul, Turkey; ^5^Department of Bioengineering, Faculty of Engineering, Marmara University, Istanbul, Turkey

**Keywords:** *Pseudomonas aeruginosa*, extremophiles, quorum sensing, biofilm, antivirulence, *Natrinema versiforme*

## Abstract

*Pseudomonas aeruginosa* is an opportunistic pathogen that causes high morbidity and mortality rates due to its biofilm form. Biofilm formation is regulated via quorum sensing (QS) mechanism and provides up to 1000 times more resistance against conventional antibiotics. QS related genes are expressed according to bacterial population density via signal molecules. QS inhibitors (QSIs) from natural sources are widely studied evaluating various extracts from extreme environments. It is suggested that extremely halophilic Archaea may also produce QSI compounds. For this purpose, we tested QS inhibitory potentials of ethyl acetate extracts from cell free supernatants and cells of *Natrinema versiforme* against QS and biofilm formation of *P. aeruginosa*. To observe QS inhibition, all extracts were tested on *P. aeruginosa lasB-gfp*, *rhlA-gfp*, and *pqsA-gfp* biosensor strains and biofilm inhibition was studied using *P. aeruginosa* PAO1. According to our results, QS inhibition ratios of cell free supernatant extract (CFSE) were higher than cell extract (CE) on *las* system, whereas CE was more effective on *rhl* system. In addition, anti-biofilm effect of CFSE was higher than CE. Structural analysis revealed that the most abundant compound in the extracts was *trans* 4-(2-carboxy-vinyl) benzoic acid.

## Introduction

Multidrug-resistant and extensively drug-resistant microorganisms arise from increased and uncontrolled antibiotic utilization (Gohil et al., 2018). It was reported that at least 2 million people are infected and approximately 23,000 people die annually due to drug resistant infections in United States (Center of Disease Control and Prevention [CDC], 2018). Treatment of infections caused by *Pseudomonas aeruginosa* can be particularly complicated due to its acquired multidrug resistance, its virulence factors and biofilm formation resulting in community-acquired and hospital-acquired infections (Singh et al., 2000; Smith and Iglewski, 2003; Bjarnsholt et al., 2010). This opportunistic pathogen causes nearly 11% of nosocomial infections especially in immunocompromised patients with cystic fibrosis (CF), pneumonia, urinary tract infections, surgical site infections, sepsis, and skin infections especially observed in burn units (Khan et al., 2015; Pires et al., 2015). *P. aeruginosa* can colonize easily in hospital equipments such as disinfectants, ventilators, food, items in bathroom and toilets. Due to its ubiquity, this bacterium can be transmitted by food, visitors, patient contact with contaminated reservoirs, healthcare worker hands, and by ingestion of contaminated materials. Unfortunately, this bacterium can also cause high mortality rates in CF patients (Ciofu et al., 2015). The Center of Disease Control and Prevention (CDC) reported that more than 6,000 (13%) of 51,000 *P. aeruginosa* infections in the United States are experienced due to multiple drug resistance each year and 400 of these infections result in death (Center of Disease Control and Prevention [CDC], 2013). According to the list of antibiotic priorities published by the World Health Organization [WHO], 2017, *P. aeruginosa* has critical requirement for new antibiotic development (Tacconelli et al., 2018). Therefore, the urgent need for alternative strategies against antibiotic resistance problem is a growing problem in the World. Recently proposed antivirulence approaches could be a powerful solution to overcome this complication (Rasko and Sperandio, 2010). The goal of these approaches is not to kill bacteria but to prevent bacterial infections and damage caused by them. In recent years, quorum sensing (QS) mechanisms are evaluated as a potential target for this strategy (Rutherford and Bassler, 2012; Tang and Zhang, 2014; Defoirdt, 2018; Dickey et al., 2017). Bacteria communicate with each other via QS by using signal molecules called autoinducers (AIs). *P. aeruginosa* employs acyl-homoserine lactones (AHLs) and has four hierarchically connected QS systems for interspecies communication: *las, rhl, pqs*, and *iqs.* The AIs produced by *las, rhl, pqs*, and *iqs* systems are 3-oxo-C12-HSL, C4-HSL, 2-alkyl4-qionolones, and 2-(2-hydroxyphenyl)-thiazole-4-carbaldehyde, respectively. The concentration of AHL molecules rises depending on bacterial population and induces activation of transcriptional regulators. Thereby, pathogenic bacteria can fight against the host immune system by the collective expression of virulence genes (Papenfort and Bassler, 2016).

Recently, several compounds and enzymes disrupting QS system have been reported. This phenomenon is called quorum quenching (QQ). Since QS systems have clinical importance in the course of pathogenesis, potential QS inhibitors (QSIs) are being still investigated. Most of these molecules have been reported from natural sources, but their synthetic derivatives are also available (Martinelli et al., 2004; LaSarre and Federle, 2013).

The term “extremophiles” is referred to microorganisms that grow in extreme environmental stress conditions. The role of extremophilic QS systems is not fully studied and remains scarce (Montgomery et al., 2013). Maheshwari and Saraf (2015) reported that halophilic bacteria produce exopolysaccharides, compatible solutes and enzymes and the synthesis of these biomolecules may be influenced by QS. Similarly, Tommonaro et al. (2012) demonstrated QS related production of diketopiperazines (DKPs) from *Haloterrigena hispanica*. The researchers reported that cyclo-(L-Pro-L-Val) induced QS system in NTL4 (AHL bioreporter strain) and they suggested that this compound may be involved in QS system of Archaea to survive in such harsh conditions (Tommonaro et al., 2012). Also, autoinducer-2 (AI-2) which provides bacterial communication for interspecies, was detected in *Halobacillus halophilus* (Tommonaro et al., 2012; Tommonaro et al., 2015). Several studies focused on such compounds examining their potential to be stabilizers of biomolecules, stress protective agents, and halophilic enzymes for biocatalysts (Finore et al., 2011; Poli et al., 2011).

Amongst extremophiles, halophiles were reported to have potential natural source with QQ activities (Teasdale et al., 2011). Quadri et al. (2016) reported that the most antimicrobial producer genus was *Natrinema*, compared to 102 extremely halophilic archaea isolated. It was reported that sublethal doses of compounds or extracts with antimicrobial activities may have anti-QS potential in the discovery of compounds with QSI potentials. In this respect, the aim of our study is to determine the presence of compounds with potential QQ and anti-biofilm activities in *N. versiforme* species belonging to *Natrinema* genus against *P. aeruginosa*. The QSI and anti-biofilm activities of ethyl acetate cell extract (CE) and cell free supernatant extract (CFSE) obtained from *N. versiforme*, an extremophilic halophile, are firstly examined against *P. aeruginosa las, rhl*, and *pqs* systems in this study.

## Materials and Methods

### Bacterial Strains

*Pseudomonas aeruginosa lasB-gfp* (Hentzer et al., 2002), *rhlA-gfp* (Yang et al., 2009), and *pqsA-gfp* (Yang et al., 2007) biomonitor strains were used to evaluate QS inhibitions and PAO1 wild type strain ATCC 15692 was used for biofilm inhibition assays. Biomonitor strains were designed for green fluorescent protein (GFP) expression under the control of P_las_, P_rhl_ or P_pqs_ promoters. They were kindly provided by Tim Holm Jakobsen and Michael Givskov from University of Copenhagen. The bacteria were normally grown in lysogeny broth (LB) medium. QSI assays were performed using M9 minimal media with thiamine (2.5 mg/l), glucose [0.5% (wt/vol)], and casamino acids [0.5% (wt/vol)]. Gentamicin was added to LB and M9 media for biosensor strains. Details of the QS monitor strains, PAO1 (wild type) and clinical isolate of *P. aeruginosa* are presented in Table 1.

**TABLE 1:** Bacteria strains used in this study.

**Strains**	**Description**	**References**
***P. aeruginosa***		
PAO1 ATCC 15692	Wild type	Holloway and Morgan, 1986
*rhlA-gfp*	Gm^*r–*30γ^; PAO1-ATCC, P*_rhlA–gfp_*(ASV)-P*_*lac*_*-rhlR-mini-Tn5 based: pMHRA	Yang et al., 2009
*lasB-gfp*	Gm^*r–*30γ^; PAO1-ATCC, P*_lasB–gfp_*(ASV)-P*_*lac*_*-lasR-mini-Tn5 based: pMHLAS	Hentzer et al., 2002
*pqsA-gfp*	Gm^*r*^; PAO1-ATCC, P*_pqsA–gfp_* (ASV)-P*_*lac*_*-lasR-mini-Tn7 based-eGFP: pMHLAS	Yang et al., 2007
Clinical isolate	Pip^*r*^; Tzp^*r*^; Caz^*r*^; Fep^*r*^; Atm^*r*^; Net^*r*¥^	This study

*^¥^Pip, Piperacillin; Tzp, Piperacillin/Tazobactam; Caz, Ceftazidime; Fep, Cefepime; Atm, Aztreonam; Net, Netilmicin.*

### Growth of Extremophiles

*Natrinema versiforme* DSM 16034 (Xin et al., 2000) was grown in 372 medium containing (l^–1^): yeast extract 5.00 g, casamino acids 5.00 g, sodium glutamate 1.00 g, potassium chloride 2.00 g, sodium citrate 3.00 g, MgSO_4_⋅7 H_2_O 20.00 g, sodium chloride 200.00 g, FeCl_2_⋅4 H_2_O 36.00 mg, MnCl_2_⋅4 H_2_O 0.36 mg, pH 7 ± 0.2, at a T 37°C. Agar plates were prepared by adding 20 g agar l^–1^.

### Cell and Cell Free Medium Extraction

After the optimal growth of microorganisms, cells and cell free medium were obtained by centrifugation at 10.000 rpm for 40 min of cultures. The extraction step was performed on cells and cell-free medium with ethyl acetate for three times in ratio 1/1 (v/v) for cell free medium (250 mL) and 1/20 (w/v) for cells (3 g). Extracts were dried at rotary evaporator at a T < 40°C. Both extracts, from cells and cell-free medium, resulted as a concentrate (Teasdale et al., 2009).

### QSI Screening

A modified method of Bjarnsholt et al. (2005) was performed for the determination of QSI potentials of ethyl acetate CE and CFSE from extremophilic *N. versiforme*. QSI screenings were performed in 96-well black microplates (Nunc, Thermo Scientific). 100 μl of M9 medium was added to each well and two-fold serial dilutions of tested extracts were made by adding 100 μl to the first well and after mixing, transferring 100 μl from first to the next well and so on. Overnight cultures of the *lasB-gfp*, *rhlA-gfp*, and *pqsA-gfp* monitor strains were added to obtain a total volume of 200 μl with an OD 450 nm of 0.1. Final concentrations of tested extracts were 240 μg/ml, 120 μg/ml, and 60 μg/ml. The tests were performed in three replicates. Bacterial growth and GFP expressions were monitored using Cytation 3 multimode microplate reader (BioTek, United States) for 14–16 h, measuring absorbance and fluorescence every 15 min. Fluorescence of GFP expression was measured at 485 nm excitation and 535 nm emission wavelengths.

### Biofilm Experiments

*Pseudomonas aeruginosa* PAO1 strain was incubated overnight at 37°C in LB and later diluted to OD 600 nm: 0.01 with M9 minimal medium including 2.5 mg/l thiamine, 0.5% (w/v) glucose, and 0.5% (w/v) casamino acid. In 96-well microplates, CE and CFSE of extremophilic *N. versiforme* were tested at the concentrations of 240 μg/ml, 120 μg/ml and 60 μg/ml. The tests were performed in three replicates. A modified protocol of George A. O’Toole (2011) was performed for biofilm staining. After incubation, the plates were washed with distilled water and then dried for 1 h at 60°C for biofilm fixation. After drying step, 125 μl of a 0.1% solution of crystal violet were added to each well. The plates were incubated at room temperature for 10 min and then washed three times. After drying, 125 μl 96% ethanol were added to the wells and absorbance were measured in a plate reader at 590 nm in microplate reader (Cytation 3-BioTek).

### Compound Isolation and Characterization

Silica gel plates were purchased from E. Merck (Darmstadt, Germany). Compounds on TLC plates were visualized under UV light. Ethyl acetate extracts were screened by thin layer chromatography (TLC) technique. Pure Compounds were recovered by preparative TLC procedure (system solvent Chloroform/Methanol 8:2 by vol).

Samples for NMR analysis were dissolved in Methanol-d_4_. Chemical shifts are reported with the residual MeOD (δ_H_ 3.32 ppm) as the internal standard for ^1^H NMR spectrometry, and MeOD (δ_C_ 49.0 ppm) for ^13^C NMR spectrometry. 1D- and 2D-NMR spectra were recorded at 600.13 MHz on a Bruker Avance III-600 spectrometer equipped with a TCI Cryo ProbeTM fitted with a gradient along the *Z*-axis, at a probe temperature of 27°C. ^13^C NMR spectra were recorded on a Bruker avance 400 spectrometer equipped with a Cryo Probe Prodigy (100.62 MHz). ^1^H,^13^C NMR, COSY, TOCSY, HSQC, HSQC-EDITED, HMBC (^3^J: 10 Hz) experiments were used for structural determinations. Mass spectra were acquired on a MicroQ-Tof mass spectrometer coupled with an Alliance HPLC (Waters, Milford, MA, United States) equipped with an ESI positive and negative source.

### Anti-virulence Assays

#### Elastase Assay

The elastase activities of *P. aeruginosa* PAO1 wild type and clinical isolate strains were determined by the protocol described by Ohman et al., 1980. 7 ml of overnight cultures (0.01 OD) were prepared in the absence or presence of *trans* 4-(2-carboxy-vinyl) benzoic acid and the cultures were incubated for 24 h with shaking at 37°C. After incubation, cultures were centrifuged at 10000 *g* for 10 min and supernatants were filtrated. 500 μl of filtrate was added to 500 μl of ECR buffer (100 mM Tris–HCl + 1 mM CaCl_2_, pH 7.2) containing 15 mg of ECR. Mixture was kept for 3 h with shaking at 37°C. Insoluble ECR was pelleted by centrifugation at 1200 *g* for 10 min. The elastase activity was evaluated spectrophotometrically at 495 nm between the *trans* 4-(2-carboxy-vinyl) benzoic acid and the untreated control groups.

#### Protease Assay

For determination of protease activity, 7 ml of overnight cultures (0.01 OD) were prepared in the absence or presence of *trans* 4-(2-carboxy-vinyl) benzoic acid and the cultures were then incubated for 24 h with shaking at 37°C. Bacteria cultures were centrifuged at 10000 *g* for 10 min and supernatants were filtrated. 500 μl of filtrate was added to 1.25% skimmed milk and mixture was kept for 30 min with shaking at 37°C. The absorbance values of each mixture were measured at 520 nm at 600 nm to determine protease activity (El-Mowafy et al., 2014).

#### Pyocyanin Assay

For pyocyanin activity, overnight cultures centrifuged at 10000 *g* for 10 min. and supernatants were filtrated. 3 ml of chloroform was added onto these filtrates. Chloroform layer was transferred to new tubes and mixed with 0.2 M HCl (Essar et al., 1990). Measurements were evaluated due to differences on OD values at 520 nm between the *trans* 4-(2-carboxy-vinyl) benzoic acid and the untreated control groups.

### *In vitro* Biocompatibility Analyses

To examine biocompatibility of samples, 24 h *in vitro* cell culture viability experiments were performed with human keratinocyte cell line HaCaT (Boukamp et al., 1988), kindly provided by Assoc. Prof. Dr. Betül Karademir from Marmara University. Cells were grown at DMEM complete media with 10% fetal bovine serum (FBS) and 1% antibiotic (penicillin, streptomycin) (PAN Biotech, Germany) at 37°C incubator with 5% CO_2_ atmosphere. When they reach confluent phase, cells were trypsinized and seeded to 96 well culture plates at the density of 10^4^ and left overnight for cellular attachment. Ethyl acetate extract fractions containing DMSO [I27 240 (1.5% DMSO) and (0.75% DMSO) 120 μg/ml, I35 and I36 (0.375% DMSO) and 60 μg/ml] were diluted in DMEM serum free media (PAN Biotech, Germany) and sterilized by passing through 0.22 μm filter and added into well plates. Cells were incubated for 24 h at 37°C with 5% CO_2_ and viability was tested with WST-1 cell proliferation kit (4-[3-(4-iodophenyl)-2-(4-nitrophenyl)-2H-5-tetrazolio]-1,3-benzene disulfonate) (Roche, Switzerland). Absorbance values at 450 nm were measured with Glomax Multi + Microplate Multimode Reader (Promega, United States). Untreated cells were used as control and amount of DMSO that samples contain were added to each sample’s control group.

### Statistical Analyses

Statistical analyses were performed by GraphPad V 5.0 Prism analyses program with Student’s *t*-test to find significant differences between samples and control group. All experiments were performed in triplicate. Data was presented as mean; biofilm error margins are presented as standard error (SE) and cell culture results are presented with 95% confidence interval (CI). A *p*-value below 0.05 was considered as statistically significant.

## Results

### The Inhibitory Effect of Extremophilic *N. versiforme* CE and CFSE on QS Systems and Biofilm Formation

Cell extract of extremophilic *N. versiforme* have more inhibitory potential than CFSE on both QS systems (*las* and *rhl*) and biofilm formation for the highest dose (240 μg/ml) applied. The inhibition ratios were recorded as 65.99% for *lasB-gfp*, 51.8% for *rhlA-gfp*, and 79.19% for *pqsA-gfp*, respectively. The same extracts at the concentration of 240 μg/ml inhibited biofilm formation by 29.57% (± 2.19).

The most effective inhibition ratios by CFSE of extremophilic *N. versiforme* on *las* and *rhl* system were obtained at the highest dose (240 μg/ml) applied. The inhibition ratios were recorded as 67.68% for *lasB-gfp*, 35.26% for *rhlA-gfp*, and 77.04% for *pqsA-gfp*, respectively. The maximum anti-biofilm effect of the CFSE extracts was determined at 240 μg/ml and the inhibition ratio was found to be 40.72% (± 4.6).

Green fluorescent protein expression graphics of the extracts with best inhibition ratios are shown in Figure 1 and biofilm inhibitions are presented in Figure 2. Inhibition data were calculated for GFP expression over absorbance values to consider bacterial growth. No significant bacteria growth inhibition was observed (Supplementary Figures S1–S9).

**FIGURE 1:**
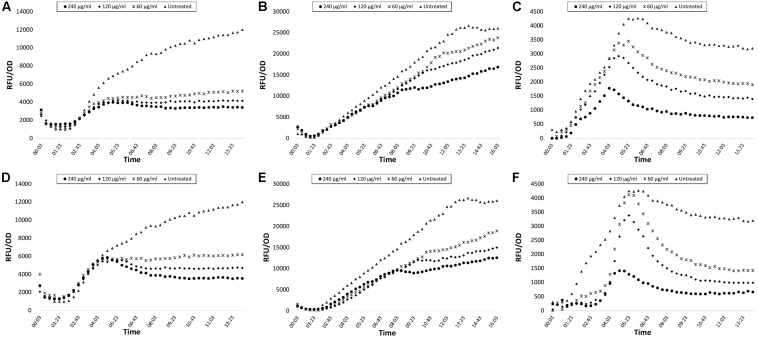
GFP expression curves of *lasB-gfp, rhlA-gfp*, and *pqsA-gfp* monitor strains treated with CFSE and CE obtained from *N. versiforme*. Data are shown as Relative Fluorescence Unit over Optical Density of 450 nm. **(A)** *lasB-gfp* CFSE, **(B)** *rhlA-gfp* CFSE, **(C)** *pqsA-gfp* CFSE, **(D)** *lasB-gfp* CE, **(E)** *rhlA-gfp* CE, **(F)** *pqsA-gfp* CE.

**FIGURE 2:**
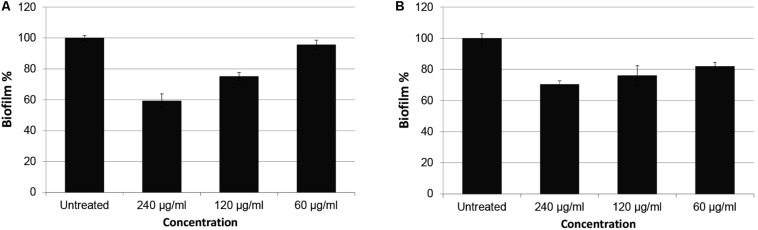
Biofilm inhibition percentages of *P. aeruginosa* PAO1 treated with CFSE and CE obtained from *N. versiforme.* Biofilm mass of untreated samples are considered as 100. **(A)** PAO1 treated with CFSE, **(B)** PAO1 treated with CE.

### Structural Characterization of Ethyl Acetate Extract

The ethyl acetate extract was preliminary investigated by TLC chromatography; it was made of almost seven UV-visible compounds present with different percentages. Furthermore, the 1H-NMR spectrum of ethyl acetate extract (Supplementary Figure S10) suggested the prevalence of a set of aromatic signals, which could be related to a unique structure, with respect to other ones.

**FIGURE 3:**
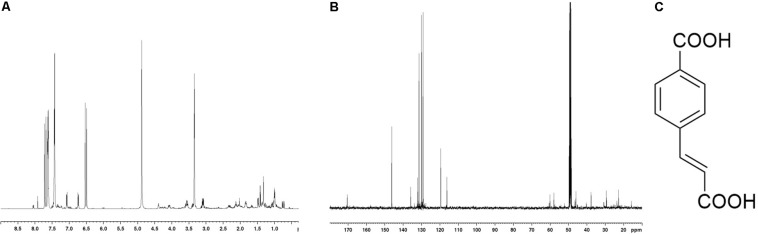
1H **(A)** and 13C **(B)** NMR spectra of *trans* 4-(2-carboxy-vinyl) benzoic acid **(C)** isolated from *N. versiforme*; spectra were recorded in Methanol-d4.

After a purification step by TLC preparative procedure, the most abundant compound (7 mg) was isolated and this molecule was spectroscopically investigated. The compound was identified as the trans 4-(2-carboxy-vinyl) benzoic acid, shown in Figure 3. 1H- 13C-NMR spectra of the isolated compound were also reported; suggesting a recovery of the molecule with a high level of purity. In 1H NMR spectrum, together with the principal signals assigned to the trans 4-(2-carboxy-vinyl) benzoic acid skeleton, the presence of very little intense signals, in particular in the aromatic and vinyl regions, suggested the scarce presence of structurally related molecules as contaminants. This structural hypothesis was confirmed by considering the presence of aromatic signals in the ^1^H-NMR at 7.06 (2H, *dd*, *5.21* and *2.10 Hz*) and 7.41 ppm (2H, *dd*, 5.21 and 2.10 Hz), which were correlated in HSQC spectrum to the ^13^C signals at 129.17 and 130.19 ppm, respectively. In HMBC experiment, long-range C-H correlations between the proton signal at 7.06 ppm and quaternary carbon signal at 131.4 ppm, and proton signal at 7.41 and quaternary carbon signal at t 135.2 ppm, secured the para substituted phenyl ring structure. Furthermore, in HMBC experiment, aromatic carbon at 135.2 ppm showed long-range correlation with a proton signal at 7.68 ppm; this last one was part of the olefin portion of the molecule. In fact, in HSQC experiment, the proton signal at 7.68 ppm (1H, d, *J* 16.10 *Hz*) was correlated at a carbon at 146.19 ppm; in addiction a further presence of an C-H olefin system was indicated by a proton signal at 6.49 ppm (1H, d, *J* 16.10 *Hz*) linked to carbon at 119.5 ppm. Long range C-H correlation between proton at 7.68 ppm and vicinal carbon at 119.5 ppm were also recorded. The presence of an intense signal at 170.41 ppm in ^13^C NMR spectrum confirmed the presence of carboxylic carbons: in HMBC experiment, a correlation with 170.4 ppm and the olefin proton signals at 6.49 and 7.68 ppm were recorded. In addiction in ^13^C spectrum, the chemical shifts of aromatic carbon at 130.4 and 135.18 ppm were typical of quaternary carbons linked to carboxylic group *para*-substituted^1^. This data suggested the presence of two carboxylic groups as reported in Figure 3. This hypothesis was confirmed by MS investigations. In ESI^–^-MS spectrum a base-peak at 146 (M-HCOOH-H^–^) confirmed this structure.

Minor compounds (0.2–0.5 mg) of ethyl acetate extract were only partially purified. ^1^H NMR spectra suggested the presence in their structure of phenyl rings (proton signals at 7.9–6.9 ppm) conjugated to alcoholic groups (proton signals at 3.5–4 ppm) or double bonds (olefin proton signals at 6.5–7.0 ppm) and/or carbonylic groups (with corresponding vicinal proton signals at 2.0–2.8 ppm). Their complete chemical characterization is still under investigation.

### QS and Biofilm Inhibition Properties of *Trans* 4-(2-Carboxy-Vinyl) Benzoic Acid

**FIGURE 4:**
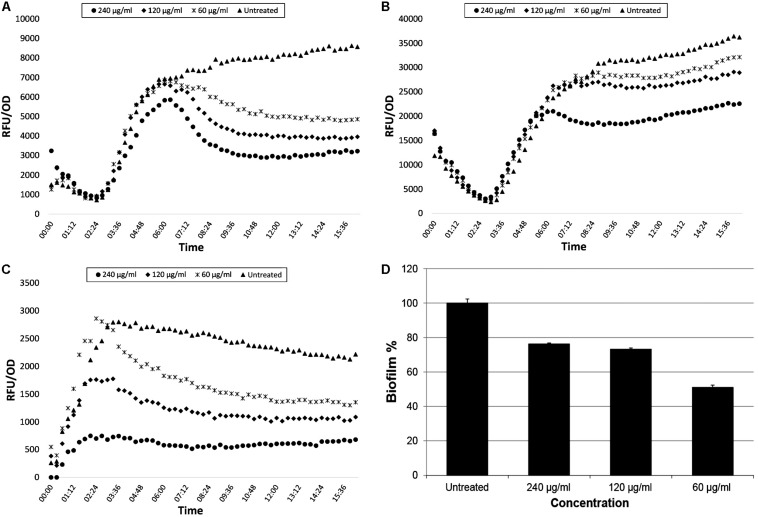
GFP expression curves of *lasB-gfp, rhlA-gfp*, and *pqsA-gfp* monitor strains (**A–C**, respectively) and biofilm inhibition percentage **(D)** of PAO1 strain treated with *trans-*4-(2-carboxy-vinyl) benzoic acid isolated from *N. versiforme*.

The isolated compound *trans* 4-(2-carboxy-vinyl) benzoic acid demonstrated similar inhibition ratios for *P. aeruginosa* QS systems as CFSE. The inhibition ratios for the concentration of 240 μg/ml were recorded as 61.85% for *lasB-gfp*, 37.71% for *rhlA-gfp*, and 74.16% for *pqsA-gfp*, respectively. Best biofilm inhibition, however, was observed at the concentration of 60 μg/ml that is 48.91%. QS and biofilm inhibition ratios of *trans* 4-(2-carboxy-vinyl) benzoic acid are shown in Figure 4.

### Anti-virulence Properties of *Trans* 4-(2-Carboxy-Vinyl) Benzoic Acid

**FIGURE 5:**
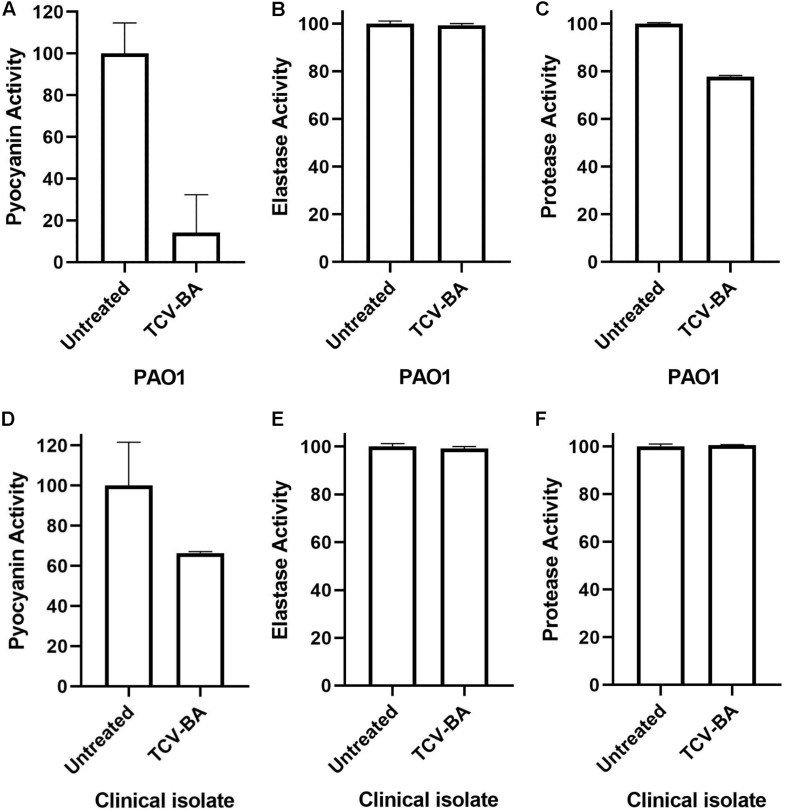
Pyocyanin, protease and elastase inhibition activities of *trans-*4-(2-carboxy-vinyl) benzoic acid (TCV-BA) isolated from *N. versiforme* against PAO1 **(A-C)** and the clinical isolate **(D–F)** of *P. aeruginosa*.

Highest inhibition was recorded on pyocyanin production of PAO1 (86%). On the other hand, the same inhibition efficacy could not be detected on the clinical isolate of *P. aeruginosa* (34.3%). Protease activity was suppressed by the compound for 27% in PAO1 whereas, 3.9% in the clinical isolate of *P. aeruginosa*. Nevertheless, elastase activity could not be inhibited by our compound in either strain. These results indicate that our compound is considerably effective for the inhibition of pyocyanin production. Results of the virulence assays are presented in Figure 5.

### *In vitro* Biocompatibility Analyses

**FIGURE 6:**
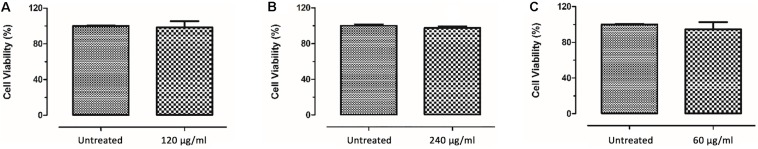
HaCaT cell viability results after incubation with CFSE **(A,B)** and *trans-*4-(2-carboxy-vinyl) benzoic acid **(C)** for 24 h.

According to safety doses of DMSO, current study estimated doses to be below 1% and concentrations of the samples are chosen due to their DMSO solvent percentages (Galvao et al., 2014). Cell viability results of HaCaT cells after 24 h incubation with CFSE and its two fractions are shown in Figure 6. Viability of cells treatedse activity was suppressed by with 120 and 240 μg/ml CFSE was 98.52% and 97.68%, respectively; while for isolated *trans-*4-(2-carboxy-vinyl) benzoic acid (60 μg/ml) it was 94.73%. Control groups were estimated as 100% viable. Statistical analyses of data showed no significance between control group and samples except CFSE 240 μg/ml, which showed difference with its control group with a *p*-value of 0.0099.

## Discussion

Recently, QS inhibition has been proposed as an antivirulence approach to overcome global drug resistance problem. This combating strategy against bacterial diseases focuses on the inhibition of bacterial virulence factors instead of their growth. Although natural QSIs and their synthetic derivatives have been reported against *P. aeruginosa*, many potential QSIs are being still investigated (Bjarnsholt et al., 2005; Skindersoe et al., 2008; Yaniv et al., 2017; Fong et al., 2018). Extremophiles are known potential sources for biotechnological and industrial aspects. Several molecules produced by Archaea and their potential role are discussed in the literature (Charlesworth and Burns, 2015). However, there is limited number of studies evaluating QQ properties or AHL degrading enzymes of extremophiles (Seo et al., 2011; Teasdale et al., 2011; Abed et al., 2013; Morohoshi et al., 2015; Santhakumari et al., 2016). Also, there is very little information about halophilic bioactive compounds with QQ potentials (Abed et al., 2013). The compounds which have been reported to have QS inhibitory potentials are cyclo (L-Pro-L-Phe) and cyclo (L-Pro-L-isoLeu) from *Marinobacter* sp. (SK-3); phenethylamides and a cyclic dipeptide isolated from *Halobacillus salinus*, *Bacillus* or *Halobacillus* sp.; palmitic acid from *Synechococcus elongatus* (Abed et al., 2013; Santhakumari et al., 2016, 2017). Taken together, our knowledge about QS system and potential QQs in extremophiles guided us to hypothesize that extremophilic microorganisms can adapt to harsh conditions by producing some other unstudied metabolites to compete with other organisms. For this purpose, we evaluated QSI and anti-biofilm potentials of extremophilic *N. versiforme* against *P. aeruginosa* biomonitor strains *lasB-gfp, rhlA-gfp*, and *pqsA-gfp*. QQ activities are generally evaluated by inhibition in the production of violacein or bioluminescence and/or reduction in biofilm formation and EPS production. The production of violacein by reporter biosensor strains (CV026 and CV017 etc.) is utilized as an indicator of QS system depending on produced AHLs by Gram-negative bacteria. However, GFP-producing biomonitor strains provide monitoring of direct gene expressions such as *las, rhl*, and *pqs*, and thus QSI potentials of tested materials can be easily evaluated on each QS system.

In our study, we detected that CE and CFSE of *N. versiforme* were considerably capable of inhibiting all QS systems (*las, rhl*, and *pqs*) at the certain concentration of 240 μg/ml. In particular, inhibition percentages in *las* and *pqs* systems were higher than *rhl* system for CE and CSFE extracts, respectively. But QS inhibition rates in *rhl* system were also observed. Our findings demonstrate consistence with other studies, because *las* system hierarchically orchestrate the expression of *pqs* and *rhl* systems. In our study, we showed inhibition in at least two QS pathways: *las* and *pqs* system. Furthermore, we analyzed anti-biofilm potentials of both extracts against *P. aeruginosa* PAO1 wild type strain and we observed that CFSE inhibited biofilm formation better than CE. Our results indicate that the inhibitory potential of CFSE of *N. versiforme* was more significant on *las* and *pqs* systems and biofilm formation whereas CE was more successful on *rhl* QS system.

We also isolated *trans* 4-(2-carboxy-vinyl) benzoic acid via a combination of chromatographic and spectroscopic techniques from CFSE. This compound is related chemically to cinnamic acid and its derivatives that are described as active antimicrobial compounds (Sova, 2012; Guzman, 2014). In particular, cinnamaldehyde have been reported to have QS inhibitory activity in bioreporter strains of *E. coli* or *Vibrio* species (Niu et al., 2006; Brackman et al., 2008). Several synthesized cinnamic acid derivatives were evaluated for their QSI potentials (Chen et al., 2019). Naik et al. (2013) reported QS inhibition by methanol extracts of marine sponge–derived actinomycetes through *Chromobacterium violaceum* CV12472. Also, they detected cinnamic acid from the most successful extract for QS inhibition. Herein, we reported the anti-QS and anti-biofilm activities of the isolated compound, *trans* 4-(2-carboxy-vinyl) benzoic acid, which is a derivative of cinnamic acid, via *P. aeruginosa* biosensor strains called *lasB-gfp, rhlA-gfp*, and *pqsA-gfp* strains and wild type PAO1, respectively.

Quorum quenching properties of benzoic acid and derivatives have been also reported against different bacteria species such as *E. coli* and *V. harveyi* (Brackman et al., 2009; Ta and Arnason, 2016). In our study, *trans* 4-(2-carboxy-vinyl) benzoic acid showed higher QS inhibitory potential than the tested extracts (CEs and CFSEs). In addition, biofilm inhibition, especially at 60 μg/ml, was found to be higher than the inhibition ratios determined for other extract concentrations. More recently, Rajkumari et al. (2018) reported the inhibitory effect of cinnamic acid on *P. aeruginosa* biofilm formation by 50.13%. In their study, authors indicated that they applied sub-MIC concentration (250 μg/ml) of cinnamic acid against *P. aeruginosa*. On the other hand, we determined that 60 μg/ml of *trans* 4-(2-carboxy-vinyl) benzoic acid is profoundly efficient for biofilm inhibition by 48.91%. Therefore, our isolated compound has potential anti-biofilm effect with low concentration. There are stringent benchmarks that have to be met by new compounds in drug discovery such as efficacy and safety criterias: broad-spectrum efficacy and no toxicity. Thus, we also performed biocompatibility tests via cell culture for 60 μg/ml pure compound, which showed no specific toxicity.

In some cases, expected effects of QSI compounds cannot be observed against clinical isolates. It is assumed that some clinical isolates of *P. aeruginosa* may have protection against various compounds (García-Contreras et al., 2014; Guendouze et al., 2017). In this respect, the effect of *trans* 4-(2-carboxy-vinyl) benzoic acid on protease, pyocyanin and elastase activities were also tested both against a clinical isolate of *P. aeruginosa* and PAO1 wild-type strain. *Trans* 4-(2-carboxy-vinyl) benzoic acid highly inhibited pyocyanin production in both strains. However, protease activity inhibition was only observed in PAO1, and no elastase inhibition was detected in either strain. In a previous study, the necessity of PqsE for production of pyocyanin was reported (Rampioni et al., 2007). In this study, we observed a high inhibition ratio against the *pqs* system, which confirms the decrease all pyocyanin production in the treated strains. On the other hand, we didn’t detect the expected inhibition for elastase.

In conclusion, we intended to show that extremophilic microorganisms can synthesize compounds that can be utilized in antivirulence approaches, specifically QS and biofilm inhibition. We investigated cell and cell-free extracts of *N. versiforme* and isolated a pure compound named *trans* 4-(2-carboxy-vinyl) benzoic acid, and all have shown QSI properties against *P. aeruginosa*. It is possible that other bioactive compounds can be isolated from other extremophilic microorganisms that live under stressful conditions. Further studies will reveal the potential use for the compound isolated in antivirulence drug research.

## Data Availability Statement

The datasets generated for this study are available on request to the corresponding author.

## Author Contributions

NS, GT, and ET designed and organized the study. AT, GA, and CI obtained and analyzed extracts. TB and BG conducted microbiological experiments. ME conducted cell toxicity experiments. TB, DB, BG, GT, ME, and NS prepared the manuscript. All authors read and approved the final manuscript.

## Conflict of Interest

The authors declare that the research was conducted in the absence of any commercial or financial relationships that could be construed as a potential conflict of interest.

## Footnotes

1 https://www.chemicalbook.com/SpectrumEN_65-85-0_13CNMR.htm
