# Supplementary material for: Extremophilic Natrinema versiforme Against Pseudomonas aeruginosa Quorum Sensing and Biofilm
Source: Front Microbiol. 2020 Feb 6;11:79. doi: 10.3389/fmicb.2020.00079 (PMC7015896; doi:10.3389/fmicb.2020.00079)
Supplement: Supplementary file 1 [file Data_Sheet_1.DOCX]

Supplementary Material

# Supplementary Figures

**Figure S1.** Bacterial growth curve of *P. aeruginosa* *lasB-gfp* monitor strain treated with CE.

**Figure S2.** Bacterial growth curve of *P. aeruginosa* *rhlA-gfp* monitor strain treated with CE.

**Figure S3.** Bacterial growth curve of *P. aeruginosa* *pqsA-gfp* monitor strain treated with CE.

**Figure S4.** Bacterial growth curve of *P. aeruginosa* *lasB-gfp* monitor strain treated with CFSE.

**Figure S5.** Bacterial growth curve of *P. aeruginosa* *rhlA-gfp* monitor strain treated with CFSE.

**Figure S6.** Bacterial growth curve of *P. aeruginosa* *pqsA-gfp* monitor strain treated with CFSE.

**Figure S7.** Bacterial growth curve of *P. aeruginosa* *lasB-gfp* monitor strain treated with trans 4-(2-carboxy-vinyl) benzoic acid.

**Figure S8.** Bacterial growth curve of *P. aeruginosa* *rhlA-gfp* monitor strain treated with trans 4-(2-carboxy-vinyl) benzoic acid.

**Figure S9.** Bacterial growth curve of *P. aeruginosa* *pqsA-gfp* monitor strain treated with trans 4-(2-carboxy-vinyl) benzoic acid.


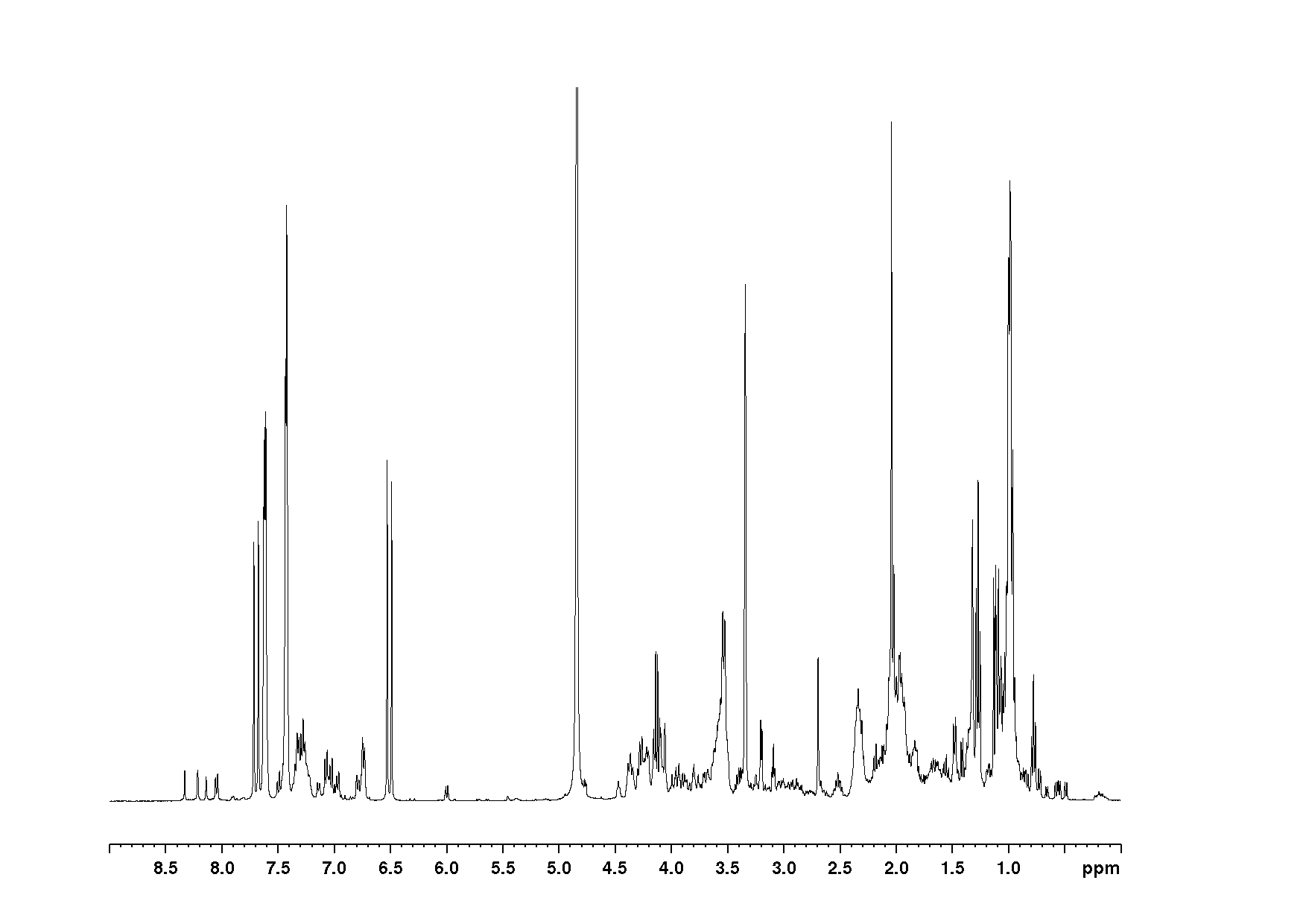


**Figure S10.** 1H –NMR spectrum of ethyl acetate fraction; spectrum was recorded in Methanol-d4.
